# The Bilingual Native Speaker Competence: Evidence From Explicit and Implicit Language Knowledge Using Elicited Production, Sentence-Picture Matching, and Pupillometry

**DOI:** 10.3389/fpsyg.2021.717379

**Published:** 2021-09-16

**Authors:** Anna-Lena Scherger, Gianna Urbanczik, Timon Ludwigs, Jasmin M. Kizilirmak

**Affiliations:** ^1^Department of Rehabilitation Sciences, Language & Communication, TU Dortmund University, Dortmund, Germany; ^2^University of Hildesheim, Institute for Psychology, Neurodidactics & NeuroLab, Hildesheim, Germany; ^3^German Center for Neurodegenerative Diseases, Cognitive Geriatric Psychiatry, Göttingen, Germany

**Keywords:** language production, language comprehension, ditransitives, implicit and explicit knowledge, article omission, pupillometry

## Abstract

The present pilot study investigated potential effects of early and late child bilingualism in highly proficient adult bilinguals. It has been shown that some early second language (eL2) speakers stagnate when it comes to complex linguistic phenomena and that they display subtle difficulties in adulthood. Therefore, we have chosen the complex structure of double object constructions. We investigate the long-term achievement in a combined-method approach using elicited production, explicit comprehension by sentence-picture matching and a measure of implicit linguistic knowledge, namely pupillometry. This eye tracking method is suitable for measuring implicit reactions of the pupils to unexpected or ungrammatical stimuli. For production, ditransitive structures were elicited by means of a game. For comprehension, a sentence-picture matching task was conducted. Two pictures were shown on a monitor that were equal with respect to the involved objects, but the thematic roles of direct and indirect objects were interchanged. Items were controlled for length, gender, animacy, semantic likelihood and word order. Reaction times and accuracy scores were analyzed. To this end, *N* = 18 bilingual adult speakers of German (+ another language, mean age: 26.5) with different ages of onset participated in this study and were compared to *N* = 26 monolingual German adult speakers (mean age 23.9). All participants had a proficiency of German above 89% correct in placement and cloze tests. Results show fully comparable productive and comprehensive competencies in monolinguals and bilinguals including the reaction times in the sentence-picture matching task and a word order effect on the reaction times in both groups. In the pupillometry task, we found monolinguals and bilinguals to be sensitive to differing conditions with respect to grammatical and ungrammatical utterances. However, we find between group differences in pupil dilations in that bilinguals react differently to strong grammatical violations than monolinguals. These results are discussed with respect to the term of native speaker competence and the variation within both groups.

## Introduction

In research on bilingual language acquisition and its outcome in adulthood, it has been shown that some of the children acquiring an early second language (eL2) are doing well on this acquisition task and others struggle with more complex structures at some point in their acquisition process and stagnate or fossilize (Paradis, 2016). Paradis (2019) discusses eL2 children's outcome and ultimate attainment in adulthood. While they are able to reach high proficiency, are typically indistinguishable from monolingual speakers in conversation, and score within the normal range of monolingual performance in most language tasks, there are subtle differences between monolinguals and bilinguals with respect to production and grammaticality judgement tasks regarding complex morphosyntax in adulthood. Such long-term achievement in (e)L2 acquisition is marked by high inter-individual variability which in turn has been attributed to age, age of onset (AOO), a broad array of external environmental and social factors (such as socio-economic status or input quality and quantity), and also to individual differences in the affective and cognitive domains influencing acquisition (Granena, 2014).

In the present pilot study, we investigated in depth highly proficient bilingual adults with various ages of onset instead of focusing on moderating factors of the ability to become highly proficient in a second language (L2). For this purpose, we performed a study with respect to the different modalities of production and comprehension and implicit grammaticality judgements, regarding ditransitive structures, a complex morphosyntactic phenomenon. Our aim was to evaluate possible subtle differences between bilingual and monolingual speakers, as Paradis (2019) postulated. The purpose of this in-depth investigation of one linguistic phenomenon was to illuminate the term “native speaker” and to evaluate whether bilinguals exhibit a level of proficiency comparable to monolinguals' performance on production, comprehension and implicit grammaticality judgement tasks.

In the literature on the definition of a native speaker, we find different approaches and different acquisition types discussed (see, e.g., the discussion on native vs. non-native foreign language learners in Clahsen and Felser, 2006) as well as varying age spans investigated. Several suggestions have been made on how to distinguish between native and non-native speakers. Amongst others, Halliday (1975) characterized a native speaker as *someone who is able to predict what the other person is going to say*, therefore enabling a native speaker to anticipate upcoming input. Others suggested to characterize native speakers by their *competence to identify ungrammatical utterances* and thus to provide valid grammaticality judgements on their language using intuitive knowledge of the grammatical sentence (Chomsky, 2002).

While there seems to be a common ground on what native speakerism means, it is in many cases unclear what it does not include. It is for instance obvious that a “bilingual is not two monolinguals in one person” (Grosjean, 1989). There is cross-linguistic influence at play in bilingual development (Müller and Hulk, 2001) that is attested in many morphosyntactic domains in 2L1 and eL2 (e.g., Schmitz et al., 2012; Scherger, 2016; for a meta-analysis see van Dijk et al., 2021). This interaction of languages within a bilingual individual is “part and parcel of bilingual development” (van Dijk et al., 2021, p. 1). However, it is unclear whether early bi- or multilingual speakers are native speakers of two or more languages (Wilkinson, 2020, p. 285). Only AOO appears to be central to the definition of native speakerism: a native speaker has acquired his or her native language from birth or at least from an early age (Davies, 2004). Nonetheless, acquiring a language at an early age is no guarantee for high proficiency, and, conversely, acquiring a language during adulthood does not rule out the attainment of high proficiency (Ortega, 2019).

Beyond that, the native speaker concept gives the impression to have “a strong monolingual bias” (Dewaele, 2017, p. 236), which results in a number of issues. Firstly, the concept has been taken to imply that monolingualism is “the gold standard” (Mauranen, 2012, p. 4), which is unsurprisingly controversial. More importantly, monolingualism is rather untypical in a predominantly multilingual world, yet monolinguals are commonly used as control groups in studies on bi- and multilingualism, reinforcing the native vs. non-native dichotomy (Dewaele, 2017). From a psycholinguistic perspective, the comparison between L1 and L2 speakers, or native and non-native speakers, respectively, is a neutral, non-judgmental comparison with the aim of gaining further understanding of how language is stored, represented and processed in the human brain.

The central issue, however, appears to lie in the term “native” itself. It is commonly perceived as a synonym for high linguistic competence or proficiency, whereas non-native speakers are often considered less competent (for a discussion see Cook, 2008 and Dewaele, 2017). It is, on the other hand, commonly accepted that there is large variation within non-native but also among native speakers when it comes to proficiency. This study, therefore, aims at investigating whether complex morphosyntactic phenomena, such as ditransitive structures, reveal a dichotomy in so-called native and highly proficient non-native speakers' productive and receptive language skills. To test this, we performed experiments using both online and offline methods, i.e., tasks that investigate language during and shortly after processing. Results are intended to shed light on the question whether non-native speakers inevitably perform below the level of native speakers in tasks involving complex grammatical structures, or whether high language proficiency, albeit non-native, is on the same level as native speakers' performance.

Turning to possible stagnation/fossilization in complex morphosyntactic phenomena in eL2 acquisition, the findings by Paradis (2016, 2019) put to question whether the phenomenon under investigation is fully mastered in bilingual acquisition. In monolingual German acquisition, ditransitive structures are the latest to be acquired regarding dative case marking (Scherger, 2015) and case marking is considered a very late acquisition phenomenon in both, monolingual and bilingual acquisition (Schulz and Grimm, 2019). To the authors' knowledge, it has not been investigated so far whether a complete mastery in eL2 bilinguals' adult outcome can be documented. Therefore, an in-depth scrutiny of ditransitives is promoted here.

Since we include highly proficient bilingual adults in our sample who had different AOOs, in what follows, we briefly present the state of research with respect to the sensitive period in bilingual language acquisition and report what previous research states about potential outcomes in adulthood. Furthermore, we introduce the linguistic phenomenon under investigation and give some background information about predictive processing and on pupillometry, which may be unknown to parts of the readership.

### Sensitive Period in Bilingual Language Acquisition

A classic topic in L2 acquisition concerns the role of AOO in achieving native-like ultimate attainment. Lenneberg (1967) put forward the controversial hypothesis of a “sensitive period,” suggesting that the grammar-learning ability in acquisition declines at some point. Since then, research has provided evidence that child L2 learners outperform adult L2 learners (e.g., Johnson and Newport, 1989). However, recent findings about child L2 learners have also shown that they do not always converge fully with native speakers (Paradis, 2019). AOO has been claimed to be the factor affecting such “*near*-native (rather than fully nativelike) attainment” (Bylund et al., 2021, p. 18); the maturation of the brain is one possible explanation for this. Studies report that some of the early learners and, in fact, every late learner perform “only” near-native-like when scrutinized in detail (Abrahamsson and Hyltenstam, 2009; Stölten et al., 2014). It was concluded from these differences between L1 and L2 that even short delays in language exposure (as in eL2 children) may have minor consequences for ultimate attainment (Bylund et al., 2021).

A further controversial issue is the exact offset of the sensitive period. In some studies, it turned out to be as early as 3;6 years (Meisel, 2018), and in others, it was found to be much later than previously assumed. Hartshorne et al. (2018) found in more than 600,000 children that the grammar-learning ability changes with age but is preserved almost into adulthood. They determined the age of 17;4 as the turning point from which this ability starts declining. With respect to our participants, this would mean that every bilingual participant with an AOO prior to 17;4 years is able to achieve native-like ultimate attainment.

### Ditransitive Constructions and Case Marking in German

In German, ditransitives are characterized by verbs, such as *geben* (to give) or *zeigen* (to show), that select a nominative subject (SUBJ), a direct object (DO) typically marked for accusative (ACC) and an indirect object (IO) typically marked for dative (DAT). Three thematic roles are assigned (Primus, 2012): Agent to the nominative, theme to the DO (see Example 1a) and recipient to the IO (see Example 1a). Thus, grammatical information is encoded morphologically, mostly on the determiner, which allows for a relatively free word order. 
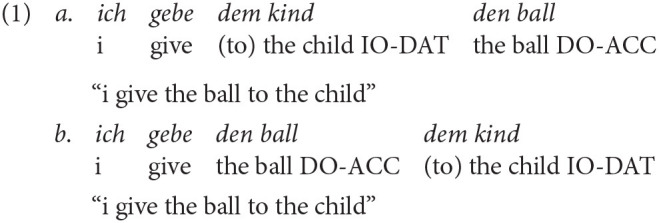


Example 1a illustrates the unmarked default word order IO-DO (Drenhaus, 2004; Kholodova and Allen, in press). While there are many instances, where DO-IO is the default word order (Müller, 1999) and may even be the underlying word order (Røreng, 2011), IO-DO is considered the unmarked word order for non-pronominal, full determiner phrases (DPs) when the IO is [+animate] and the DO is [-animate]. This word order corresponds to the canonical structure of *recipient*-*theme* proposed by Kholodova and Allen (in press), as recipients are typically [+animate]. However, when the IO is [-animate] and the DO is [+animate] the unmarked word order is DO-IO (Müller, 1999). When both objects are [+animate], IO-DO is considered the unmarked word order (Müller, 1999). The marked order for [+animate] IOs and [-animate] DOs is illustrated in Example 1b. This word order is grammatical, but rather uncommon in German.

Sauerman and Höhle (2018) showed that in child-directed speech, IO-DO is the most frequent order. As factors influencing the word order, they documented (i) animacy (animate vs. inanimate), (ii) definiteness (definite vs. indefinite), (iii) givenness (given vs. new), and (iv) reference expression (pronoun vs. full lexical phrase; Sauerman and Höhle, 2018). Furthermore, Drenhaus (2004) found effects of word order on the correct case marking in monolingual children from age 3;9 to 6;8. Word order and case marking were correctly repeated in sentences with the default word order. However, the non-default DO-IO could not be repeated correctly.

In first language (L1) and eL2 acquisition literature, there is consensus that, owing to its high complexity, German case marking is acquired very late compared to other acquisition phenomena (Schulz and Grimm, 2019). However, the exact age of mastery of this complex morphosyntactic phenomenon in different types of acquisition (Scherger, 2016, 2018; Ulrich et al., 2016; Lemmer, 2018; Schulz and Grimm, 2019) and possible fossilization of eL2 acquisition (Scherger, 2019) is still under debate. In monolingual acquisition, the reported age of mastery of the case marking paradigm is between 4;6 (Schmitz, 2006) and 9;0 years (Ulrich et al., 2016). For bilingual children, we find early primary school age as the age of mastery for simultaneous bilingual (2L1) children (Scherger, 2016), but for eL2 children, till date, no possible complete acquisition of the case paradigm has been reported. Regarding dative case marking, we know that language acquisition in eL2 children is delayed compared with L1 and 2L1 children (Lemmer, 2018; Scherger, 2019). Furthermore, prepositional case marking seems to be the case subtype acquired earliest in L1 and eL2 children (Lemmer, 2018; Scherger, 2021). Dative case marking in ditransitive structures has been shown to be the most difficult to acquire in monolingual and bilingual children's production (Scherger, 2015); therefore, these are supposed to be the most complex case structures in German matrix clauses. This is why we focus on double-object constructions with ditransitive verbs in this paper.

Before achieving target-like utterances like in Example 1, L1 children show an extended period of difficulties with these structures (Eisenbeiss et al., 2006; Schönenberger et al., 2011). Typically, nominative is acquired first, followed by accusative and dative (Eisenbeiss, 2002). Error patterns are article omission (see Example 2a by Scherger, 2015) and overgeneralization of the accusative in dative contexts (see Example 2b by Scherger, 2015). In Example (2b), there is syncretism as the definite article of feminine singular nouns is identical for nominative and accusative forms (both *die*). Here, the production of the IO *die schnecke* is assumed to be an overgeneralization of the accusative. 
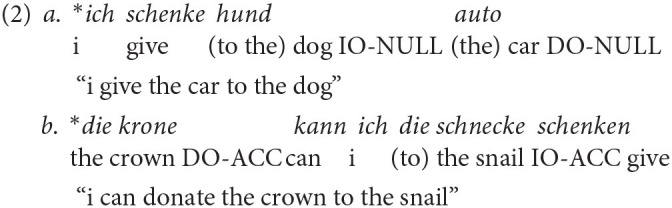


Regarding ditransitive structures, there are even fewer studies on the acquisition of comprehension capacities than on production. A related study, though not on ditransitives, was conducted by Dittmar et al. (2008). They investigated whether 2-, 5-, and 7-year-old German children are able to use the grammatical cues of case marking and word order in transitive constructions to identify agents and patients (see example 3; Dittmar et al., 2008, p. 1155). 
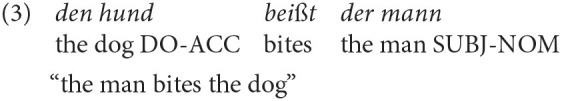


The results showed that younger children relied predominately on word order to interpret the sentences (i.e., the dog bites the man), whereas seven-year-old children behaved like adults by relying on case markers rather than word order (i.e., the man bites the dog). In line with these results, Brandt et al. (2016) report in a pointing study that older children (aged six) relied on case marking, whereas 3- and 4-year-old children employed only word order to interpret simple transitive sentences.

In an experiment using the same methods as the present study, Scherger et al. (submitted)^1^ investigated 5–7 year-old L1 children with regard to production and comprehension of ditransitives. They found that only 56.0% of these monolingual children could produce and 62.5% could comprehend as accurately as L1 adults. The results of the reaction times (RTs) in the comprehension task showed that children by that age did not react explicitly by button press in the sentence-picture matching task before hearing the second object. Monolingual adults on the other hand reacted even before the ditransitive structure's second object was auditorily presented. This was interpreted as an explicit sign of predictive processing.

### Predictive Sentence Processing in L1 and L2

An implicit reflection of language processing is the prediction of upcoming input. Earlier research on predictive processing has shown that the human parser of adult native speakers predicts upcoming input before it is heard. In this context, prediction is defined as “pre-activation/retrieval of linguistic input before it is encountered by the language comprehender” (Huettig, 2015, p. 122). For instance, in an eye tracking study Altmann and Kamide (1999, p. 250) showed that on hearing a sentence like –“The boy will eat the cake,” adult participants looked at the edible object out of the four objects presented already after hearing the verb “to eat,” thus showing anticipatory eye movements. Instead, on hearing a sentence like “The boy will move the cake,” there were no anticipations found in that there were no saccades to the cake on hearing the verb *move*. The same anticipation mechanism was found with respect to the morphosyntactic cue of case marking. Adult native speakers of German anticipated the second nominal phrase (NP) of a sentence on hearing the case-marked first NP (see Example 4; Kamide et al., 2003, p. 41). 
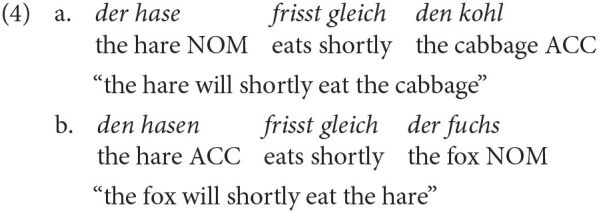


However, till date, research on adult L2 learners has shown mixed results (Kaan, 2014). Regarding transitive structures, Hopp (2015) found adult late L2 speakers in contrast to L1 speakers not to be able to use the case marking cue to predict upcoming input. Further evidence of late L2 learners not showing predictive processing (e.g., Martin et al., 2013) resulted in the hypothesis of Reduced Ability to Generate Expectations (RAGE) for late L2 learners (Grüter et al., 2014, 2017). Recently, the RAGE hypothesis was put to test for highly proficient Russian late L2 learners of German (Schlenter, 2019), investigating ditransitive structures like in example 5 (Schlenter, 2019, p. 120) in an eye tracking paradigm. 
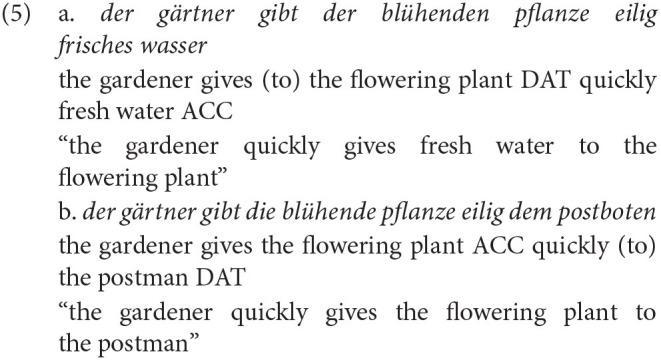


Schlenter (2019) showed that adult speakers (L1 as well as late L2) could predict the second object through anticipatory looks before hearing it. However, note that Russian has a morphologically rich case-marking paradigm that is in many ways comparable with German. Therefore, the questions of whether Schlenter's findings can be confirmed with other L1s than Russian and whether the findings remain stable when animacy is more narrowly controlled still remain unanswered.

Additionally, a recent study on anticipatory mechanisms in early bilingual language processing by Desideri and Bonifacci (2018) even found a bilingual advantage in anticipating upcoming input. They investigated bilingual Italian-German adults from Merano, where both Italian and German are official languages. Inclusion criteria required participants to have bilingual parents and an AOO before school. Therefore, we assume these speakers to be 2L1 or eL2 bilinguals. In the experiment, participants saw four pictures on a screen and had to complete an Italian sentence such as “The woman will spread the butter on the…” by pressing the key corresponding to the matching picture (e.g., flower or bread). Bilinguals were found to outperform Italian monolinguals in this sentence completion task by showing faster RTs in choosing the target word.

### Pupillometry

Pupillometry is the study of changes in pupil diameter. They are “small-scaled, rapid fluctuations in pupil diameter […] difficult to detect by unaided observation” (Beatty and Lucero-Wagoner, 2000, p. 143). However, with the advent of automatic eye trackers, which measure pupil size between 30 and 1,000 times per second, this observation has become possible. Besides the explicit responses to language described above, we are also interested in implicit signs of reactions to language input. Therefore, we use pupillometry as this method does not require explicit behavior but still identifies responses to language and acts like “a window to the preconscious” (Laeng et al., 2012).

The pupil's diameter is inherently variable with a typical size of ~3–4 mm and a range from 1 to 9 mm (Beatty and Lucero-Wagoner, 2000; Sirois and Brisson, 2014). The pupil reacts by constriction or dilation to stimulations in ~200 ms (Davson, 1972; Sirois and Brisson, 2014), e.g., to stimulations like varying luminance levels (Hepach and Westermann, 2016). More importantly, also other elements can influence the pupil size. Since the 1960s, research has repeatedly shown that factors such as arousal (Bradshaw, 1967; Bradley et al., 2008), emotion (Partala and Surakka, 2003; Zheng et al., 2014), attention (Karatekin, 2004), memory (Kahneman and Beatty, 1966; Papesh et al., 2012; Johnson et al., 2014), cognitive load/intensity and mental effort (Beatty, 1982; Porter et al., 2007; Piquado et al., 2010), novelty (Naber et al., 2013), and task complexity (Schluroff, 1982; Kosch et al., 2018) influence the pupil size, without the participants' knowledge (Laeng et al., 2012; Sirois and Brisson, 2014; Schmidtke, 2018; Zekveld et al., 2018).

As early as Hess and Polt (1964) measured pupil size in relation to simple multiplication tasks. Adult participants mentally calculated the product of small numbers in four tasks of varying difficulty (e.g., 7 × 8 = ?, 8 × 13 = ?, 13 × 14 = ?, 16 × 23 = ?), while changes in their pupil sizes were measured. The participants' pupil diameters increased with the increased difficulty of the calculation, indicating that task-evoked responses in the pupils are an effective way to measure processing load/effort (Beatty, 1982). Another paradigm that also focused on the brain's ability to create predictions or expectations is the so-called violation of expectation (VoE) paradigm. Here, pupillometry is used to identify surprise. When the participants' expectations of something are violated, pupils dilate (such as a violation of an expectation of a specific expected rhyme pattern; see, among others, Satterthwaite et al., 2007; Preuschoff et al., 2011; Yu, 2012; Scheepers et al., 2013; Lawson et al., 2017; Renner and Włodarczak, 2017).

With respect to linguistics, literature reports the use of pupillometry mostly in adults (Just and Carpenter, 1993; Scheepers and Crocker, 2004; Engelhardt et al., 2010; Fernandez et al., 2018; for a review, see Schmidtke, 2018), but to some extent also in infants and toddlers (Tamási et al., 2017; Süss et al., 2018). Scheepers and Crocker (2004) investigated syntactic priming with respect to case marking of subjects and direct objects regarding the subject-first preference in native German young adults (also called N1 bias, Lidzba et al., 2013). Scheepers and Crocker (2004) used pupillometry to identify garden-path effects employing abiguous structures. They report that structures that were disambiguated toward object-initial reading were harder to process than those disambiguated toward subject-initial reading. Pupil sizes have been found to increase with processing difficulty. Moreover, in a word recognition paradigm, Schmidtke (2014) investigated adult English monolinguals as well as Spanish-English early and late bilinguals (early bilinguals had an AOO before age 8 and late bilinguals had an AOO of 18 or later) with respect to their word retrieval effort. Pupil size was recorded while hearing an English word and matching it to one out of four pictures. Bilingual speakers displayed an overall delayed pupil response compared to monolinguals. Within the bilingual group, higher English proficiency was linked to an earlier response of the pupil. Thus, pupillometry was able to identify implicit word retrieval effort that differed between monolinguals and bilinguals.

With respect to VoE, pupillometry has been shown to be a useful marker for the ability of 30 months-old toddlers to differentiate between correct and incorrect pronunciations (Tamási et al., 2017), as well as 30- to 36-months-old children's sensitivity to attributive gender marking (Süss et al., 2018). As a sign of VoE, they demonstrated bigger pupil dilations in response to ungrammatical (^*^da ist ein blauer Haus, ‘there is a blue_MASC_ house_NEUTR_’) than to grammatical utterances (*da ist ein blaues Haus*, ‘there is a blue_NEUTR_ house_NEUTR_’).

In sum, the core findings of pupillometry-based studies on language are that it is a valuable and valid measure of linguistic complexity. The more complex a linguistic structure, the more difficult it is to process. The more surprising an upcoming input structure is, the higher the difficulty to process, and the higher the proficiency of a speaker, the less effortful the processing of particular structures. With all of these methodological advantages regarding pupillometry in mind, we tested monolingual and bilingual adults' implicit sensitivity to grammatical violation.

### Research Questions and Hypotheses

The present study investigates the questions of whether highly proficient bilingual speakers of German with various L1s reach comparable performance levels like monolinguals. Specifically, we want to answer the following questions:

Do bilingual speakers

1. produce comparable ditransitive structures to monolinguals (production),
2. comprehend ditransitive structures accurately (comprehension),
3. use the case marking cue of the first object for anticipating the thematic role of the second object (predictive processing) and
4. react implicitly to ungrammatical auditory stimuli by a change in pupil size (implicit sensitivity to grammatical violations)?

Considering prior findings, we assume that the participating L1 and 2L1 speakers can produce and understand the tested ditransitives. Therefore, we expect performance at ceiling in the production and comprehension tasks. However, we expect subtle difficulties in eL2 and late L2 learners of German regarding production of the dative case marking, because prolonged difficulties in children have been shown in Lemmer (2018) and Scherger (2019). As comprehension precedes production in acquisition (Scherger et al., submitted)^1^, we do not expect any of the investigated (highly proficient) speakers to show difficulties with comprehension accuracy. However, as difficulties in anticipatory processing within late L2 learners (Grüter et al., 2014; Hopp, 2015) and fossilizations within complex morphosyntactic domains have been reported (Paradis, 2016), we expect the eL2 and late L2 speakers to show slower RTs than 2L1 and L1 speakers. Based on findings of bilinguals outperforming monolinguals in anticipating upcoming input by Desideri and Bonifacci (2018), we even expect faster RTs in the sentence-picture matching task for 2L1 bilinguals than for L1 speakers. With respect to the implicit sensitivity to grammatical violation, we expect monolingual and bilingual speakers to behave alike. We assume that the highly proficient participants of the present study should have built grammatical representations of the target structure and therefore show comparable pupil responses, when their expectations of grammatical structures are violated. We therefore do not expect differences between monolingual and bilingual participants in the implicit grammaticality judgement task.

## Methods

### Participants

In this study, 44 adult speakers participated (see Table 1). Monolingual (*N* = 26) and bilingual (*N* = 18) participants were matched based on age (Mann-Whitney U: *U* = 511.0, *p* = 0.075). All participants were university students and received course credits for their participation in the experiments.

**Table 1:** Participants' characteristics.

**Monolinguals (*****N*** **=** **26)**			
Proficiency level		<90% correct in placement test and cloze test (overall mean = 97.0%, ***SD*** = 2.2%)	
	**Mean**	**SD**	**Range**
Age	23.9	7.6	19–48
**Bilinguals (*****N*** **=** **18)**			
Proficiency level		<89% correct in placement test and cloze test (overall mean = 96.4%, *SD* = 2.4%)	
	**Mean**	**SD**	**Range**
Age	26.5	6.14	19–38
AOO German	4;1	4.14	0;0–13
LoE in years	22.6	4.9	16.5–33

*AOO, age of onset; LoE, level of education*.

Monolingualism was defined as having acquired only one language (i.e., German) in the first years of life. This does of course not exclude the learning of an L2 at school. In the bilingual group, simultaneous and sequential bilinguals were included on purpose so that different AOOs could be compared (2L1: *N* = 6, AOO = 0;0; eL2: *N* = 7, AOO = 3;0–4;0; late L2 learners: *N* = 5, AOO = 6;0–13;0). Various L1s were included in the bilingual group: Albanian (*N* = 2), Arabic (*N* = 1), English (*N* = 1), Italian (*N* = 1), Kurdish (*N* = 1), Polish (*N* = 4), Russian (*N* = 2), Serbian (*N* = 1), Spanish (*N* = 3), Turkish (*N* = 1), and Vietnamese (*N* = 1). Participants were assigned to the corresponding groups according to our definition of mono- or bilingualism based on self-reports.

To evaluate the participants' German proficiency, we conducted a placement test with multiple-choice questions. Alternatives were manipulated regarding syntactic, lexical, semantic and pragmatic knowledge of German. Additionally, the participants completed two cloze tests, in which lexical, semantical and orthographical knowledge was tested. These were taken from the International study center of the University of Kassel, Germany. Each text contained about 70 words and was truncated canonically, i.e., starting with the second sentence, every second word's second half was truncated. For further evaluation of the participants' proficiency, three one-minute verbal-fluency tasks were carried out (Friesen et al., 2015; Lemmerth and Hopp, 2018). In the category-fluency task, participants were asked to name “animals” and “objects at home.” In a letter-fluency task, they were asked to name words starting with the letter “s.” Furthermore, we screened WM components by assessing FW and BW digit spans (WISC-V; Wechsler, 2017).

Table 2 summarizes the German proficiency and shows that no significant differences existed between monolingual and bilingual speakers. Additionally, Table 2 lists the WM results (digit spans), which also show no group differences.

**Table 2:** Participants' German proficiency.

	**Placement test**	**Cloze test**	**Letter fluency test**	**Animals**	**Objects at home**	**Overall fluency**	**Digit span FW**	**Digit span BW**
**Monolinguals**								
Mean (%)	97.3	96.8	17.1	25.9	27.7	70.7	6.5	6.1
SD (%)	2.2	3.4	4.8	6.2	6.8	17.8	1.2	1.2
Range (%)	90–100	92–100	10–26	13–39	14–39	33–95	5–10	4–8
**Bilinguals**								
Mean (%)	97.0	95.7	20.3	26.8	29.1	76.2	6.3	5.3
SD (%)	2.7	4.0	5.4	6.6	6.4	15.9	1.1	1.4
Range (%)	92–100	87–100	10–32	15–46	18–47	55–125	3–8	3–8
Mann-Whitney U test	*p* > 0.05	*p* > 0.05	*p* > 0.05	*p* > 0.05	*p* > 0.05	*p* > 0.05	*p* > 0.05	*p* > 0.05

*FW, forward; BW, backward*.

### General Procedure

Participants were tested individually in a quiet room, after providing informed consent. For the comprehension task and the pupillometry experiment, viewing distance and head position were held constantly at 70 cm by a forehead and chin rest. As prior literature reported priming effects from comprehension on production but no priming effects from production on comprehension (Kauschke and Siegmüller, 2010), we conducted the production prior to the comprehension task. Finally, we conducted the additional pupillometry experiment. Overall, a test session lasted about 60 min.

## Study 1 – Production

### Production Study Design

For eliciting ditransitive constructions, we asked the participants to play a card game with three stuffed animals (each belonging to a separate German gender: *der hund*_MASC_ [the dog], *die schnecke*_FEM_ [the snail] and *das schaf*_NEUTR_ [the sheep]). Overall, the game consisted of 27 cards with pictures of animals. The participants had to give/donate the animals on the picture cards to one recipient (one of the three stuffed animals) describing their action. This resulted in utterances like *ich gebe das pferd dem schaf* or *ich gebe dem schaf das pferd* (“I give the horse to the sheep,” see Supplementary Table 1 for further details), where both the DO and the IO were [+animate]. There were two practice examples in which the experimenter picked a card and gave it to a stuffed animal producing an example utterance. The experimenter used IO-DO in his/her examples.

To exclude confounding factors, we controlled for animacy and included only [+animate] direct and indirect objects (see Gamper, 2019, for case-animacy coalitions), all of which were animals to exclude influence of animacy hierarchy. To avoid the participants' use of semantic cues for the assignment of thematic roles, all items used were semantically reversible. In sentences like those given in Example 1, the decoding of case markings is not necessary, since semantic cues assure the assignment of thematic roles (the child cannot be given to the ball, but vice versa). Furthermore, definiteness was controlled for in the production task by using only definite nouns in full lexical DPs in the practice examples given by the experimenter. This was supposed to make the participants avoid indefinite articles and pronouns in their own productions as well. Finally, to avoid verb bias, we included two verbs (*jemandem etwas geben* and *jemandem etwas schenken*, “to give something to somebody”). These two verbs are comparable with respect to their semantics, lengths, and subcategorization frames. Both are frequent and attested to be acquired early in German (Grimm and Doil, 2006).

### Production Data Analysis

For the analysis of the production task, target-like accusative and dative case markings were counted separately (raw scores and percentages for each). Moreover, utterances were analyzed separately with respect to the word order produced: DO-IO (see Example 6a) or IO-DO (see Example 6b). 
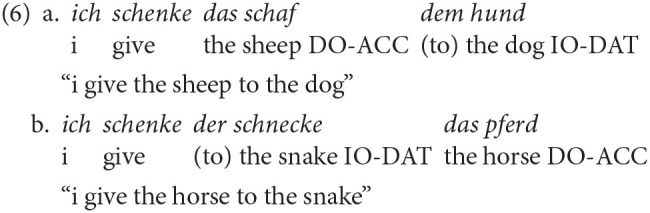


The amount of analyzed utterances in bilinguals was *N* = 462 and in monolinguals was *N* = 644 (total amount: *N* = 1,106).

On average, 1.9% (*SD* = 1.0) and 1.8% (*SD* = 0.8) of all the produced objects were built with pronouns by bilinguals and monolinguals, respectively. These occurrences were excluded from the analysis for a better match within the comparison of comprehension and production data. Furthermore, we excluded realizations of indirect objects by prepositional phrases (PPs), since this structure does not mandatorily require a dative case marking (see Example 7). Utterance dropout rates because of producing PPs were 3.3% (15/462) in bilinguals and 2.6% (17/644) in monolinguals. These numbers confirm results reported by Kholodova and Allen (in press) that participants more often realized double objects as DP-DP than as DP-PP structures. Since monolingual adults also produced these structures, they cannot be claimed non-target like (however, see Baten and De Cuypere, 2014, for a different perspective on ungrammatical PP structures). For a bilingual's example, see Example 7a and for a monolingual's example, see 7b. 
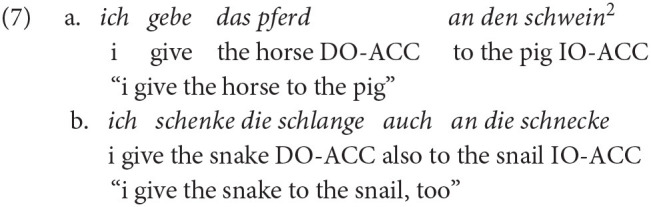


Moreover, utterances including verbs other than *geben/schenken* (to give) were excluded from the analysis since they may select different subject and object structures with different case assignments (see, e.g., *gehen an* [to go to] in Example 8). 
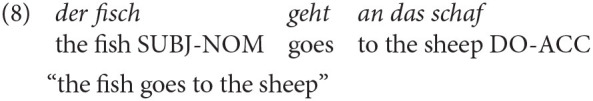


Data were analyzed using generalized linear mixed models (GLMMs) with a logit link function, assuming a binomial error distribution for the binary response (correct/incorrect production). For the analysis, we used lme4 package (Bates et al., 2015) in R version 3.6.2 (R Core Team, 2020) and RStudio version 1.2.5033 (RStudio Team, 2020). Accuracy was analyzed split for the production of the accusative and dative objects. First, we set up a model space with all reasonable models, including a null model, just including *Participant* as a random intercept. Then, models were compared using the Akaike Information Criterion (AIC^3^). For the purpose of model comparisons, all models were computed using the Maximum Likelihood estimation method. The statistics of the winning model (i.e., best performing model according to AIC) were reported in full.

To evaluate whether we should include a factor *Group* dissociating

- monolingual vs. bilingual participants only,
- participants according to their age of German acquisition onset: from birth as L1 (monolinguals), from birth as 2L1, early L2 onset, late L2 onset,
- participants based on the L1 case systems similar or different to German: same (monolinguals), similar, different,

we compared three GLMMs including different versions of *Group* as a fixed-effects factor, plus random-effects factor *Participant* as random intercept.

When categorizing the languages, we focused solely on marking of objects in the form of common nouns. Pronominal forms were not considered as only the use of full DPs (with definite articles) was relevant in this production task as well as in the following experiments (study 2 and study 3). We categorized a language as similar to the German case system when a case system with different cases used for subjects and objects as well as for the two objects of ditransitive structures was present. The categorization of case systems' similarity was specifically based on the presence of dative structures like in the German case system. All case systems of the languages categorized as similar, namely Albanian, Polish, Russian, Serbian and Turkish, have five to seven grammatical cases, among which are the accusative and the dative case. In Arabic, there are three grammatical cases. However, unlike in German, only the accusative is used for the object of a verb and it can mark both objects in double object constructions (Mohamed, 2013). Therefore, Arabic was classified dissimilar. English, Italian, Kurdish, Spanish, and Vietnamese were also categorized as dissimilar.

### Production Results

To evaluate the question whether *Group* and *WordOrder* had an influence on production accuracy, we ran separate GLMMs for produced accusative and dative objects.

#### Accusative Production Accuracy

As described under 3.2, we first evaluated the different ways to model *Group*. The GLMM performing best was the one where bilinguals were further split according to AOO (AICs: 130.51 for mono-/bilingual split vs. 130.30 for age of acquisition onset split vs. 130.37 for case system split), but the differences between models were rather small. We continued with *Group* as split by AOO.

Second, we tested the influence of *Group* as defined above and *WordOrder* {IO-DO, DO-IO} on accuracy of the accusative object. To this end, we set up a model space as reported in Table 3. The winning model was the null model M0. The random intercept for *Participant* was highly significant [odds ratio = 25069.66, 95% CI = [507.19, 1239166.04], *p* < 0.001].

**Table 3:** Model space for accuracy in the production task.

**Model**	**Formula**	**AIC**	
		**Accusative**	**Dative**
M0	Accuracy ~ 1 + (1|Participant)	**128.71**	93.80
M1	Accuracy ~ 1 + Group + (1|Participant)	130.30	95.64
M2	Accuracy ~ 1 + WordOrder + (1|Participant)	129.39	**93.63**
M3	Accuracy ~ 1 + Group + WordOrder + (1|Participant)	133.53	95.54
M4	Accuracy ~ 1 + Group + WordOrder + Group*WordOrder + (1|Participant)	-	97.36

*Formulas for all models with AICs, i.e., estimators of model fit for accusative and dative case marking accuracy, respectively. Winning models' AICs are printed in bold letters. Model 4 could not be estimated for accusative due to an error in the Hesse matrix (1 negative eigenvalue), suggesting that there was too little variance in the data for a third term in the model—as to be expected from the vanishingly low error rate*.

In other words, neither *Group*, that is, AOO, nor *WordOrder* helped explaining the variance in the data. The data were best explained by inter-individual variation only. This finding is not surprising given that monolinguals and bilinguals showed ceiling performance.

The few occurrences of errors in production within the bilingual group (see Table 4) were consistently accusative overgeneralizations in obligatory dative case contexts (see Example 9). 
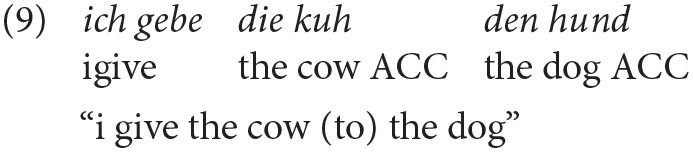


**Table 4:** Performance of ditransitives in monolinguals and bilinguals in the production task.

	**Accusative**		**Dative**	
	**IO-DO**	**DO-IO**	**IO-DO**	**DO-IO**
**Monolinguals**				
Mean (%)	100	100	100	98.9
SD (%)	0.0	0.0	0.0	3.0
Range (%)	100–100	100–100	100–100	87.5–100
**Bilinguals**				
Mean (%)	100	97.3	100	99.6
SD (%)	0.0	8.7	0.0	1.6
Range (%)	100–100	65–100	100–100	93.7–100

#### Dative Production Accuracy

Again, we first evaluated how to best model the factor *Group*. The comparison of the monolingual/bilingual vs. AOO vs. case marking system of L1 models revealed that again AOO was the slightly better estimate (AICs = 95.79 vs. 95.64 vs. 95.77). Thus, we continued with Group as AOO.

Then, we tested the influence of *Group* as defined above and *WordOrder* {IO-DO, DO-IO} on accuracy of the dative object with the same models as for the accusative (see Table 3). Surprisingly, the model M2, containing a fixed-effects factor for *WordOrder* slightly outperformed the null model M0. The random intercept of *Participant* was highly significant [odds ratio = 7175.65, 95% CI = [93.74, 549265.32], *p* < 0.001]. However, even though slightly contributing to explaining the variance observed in the data, main effect of *WordOrder* did not reach significance [β = 1.412, SE =1.065, odds ratio = 4.10, 95% CI = [0.51, 33.03], *z* = 1.326, *p* = 0.185]. It is conceivable that this is due to the very low number error rates, thus, little variance in the data.

Thus, while *Group* had no influence on dative production accuracy, *WordOrder* had a descriptive, but non-significant influence.

## Study 2 – Sentence-Picture Matching Task (Comprehension)

### Comprehension Study Design

This tasks' stimuli were presented on a standard desktop computer running Windows 10. We used a 24” flatscreen monitor with a resolution of 1366 × 768 pixels and a frame rate of 60 Hz. The experimental presentation was conducted using a video created with Microsoft PowerPoint 2016.

**Figure 1:**
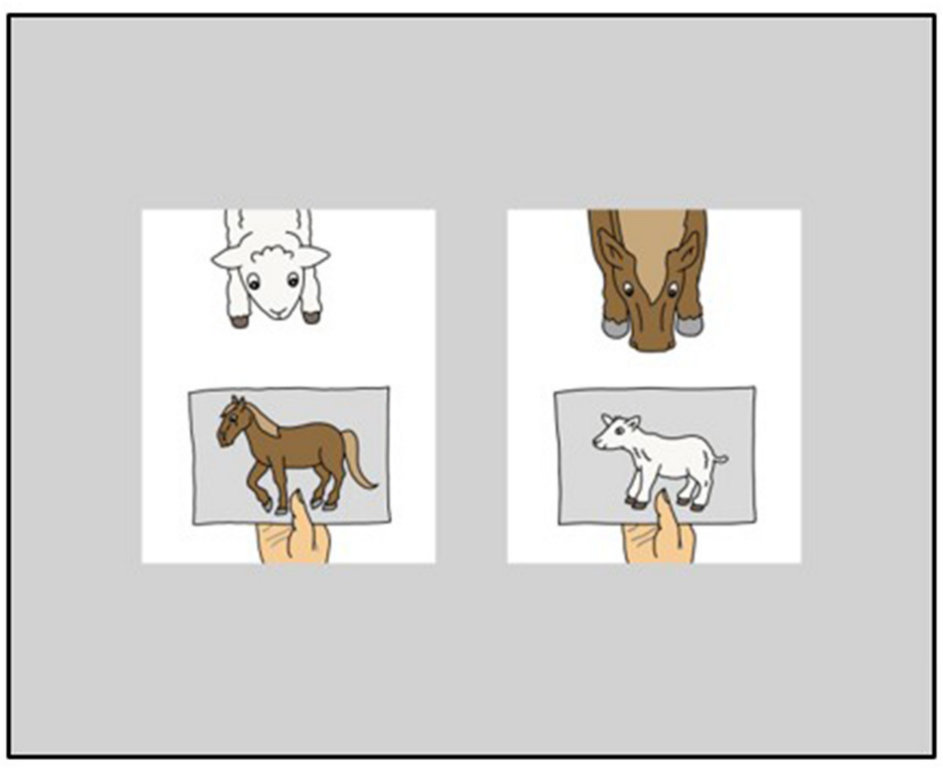
Item example as presented during the sentence-picture matching task “ich gebe dem lamm sicherlich das pferd” (“certainly, i give the horse to the lamb”).

Two pictures (408 × 491 pixels each) were simultaneously presented to the participants at a distance of 100 pixels in the middle of the screen on a gray background (see Figure 1). Participants heard a pre-recorded ditransitive construction via video (see Example 10), and were asked to press one of two buttons as soon as they had identified the matching picture: the left (key “a”) or right button (key “6” of the number block) corresponding to the side of the matching picture on screen. These buttons were chosen, because these keys are at a comfortable distance on a German QWERTZ keyboard with a number block. Both keys were marked with white stickers.

Regarding the stimuli of the comprehension task, we controlled for animacy, definiteness and verb bias as was described above for the production task. We only included objects that were [+animate], definite and consisted of full lexical DPs. To control for auditory length in addition, we restrained the maximal word length of objects to two syllables (most animals were monosyllabic). The subjects were kept consistent in 1st person singular for test trials. Regarding grammatical gender, we excluded the feminine gender from the comprehension task because of the homonym form of *der* for dative feminine singular and nominative masculine singular; since the latter is the default, the dative feminine *der* could be biased. We further excluded masculine items because of the difficulty to discriminate accusative and dative forms of *den* and *dem*. Therefore, all the objects included in the sentence-picture matching task were of neuter gender (*das schwein* [the pig], *das pferd* [the horse], *das schaf* [the sheep], *das pony* [the pony], *das lamm* [the lamb]). In addition, we added a task-irrelevant adverb between the indirect and direct object to provide time to parse the first object and react before hearing the second object. Example 10 illustrates the item composition. Figure 1 presents examples of pictures, one of which had to be chosen for this item (target picture on the left, distractor on the right). 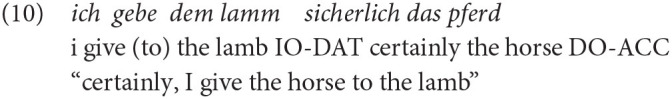


The experiment contained 58 items (20 ditransitive experimental trials and 38 fillers; e.g., *die giraffe frisst auf der wiese* [the giraffe eats in the meadow] vs. *das schaf frisst auf der wiese* [the sheep eats in the meadow], see item list in Supplementary Table 2). Prior to the experiment, four practice items were given. The experiment started only after the participants understood the task. We created two lists of items. Each list contained all experimental trials and all fillers but their order was different. The two different lists of pseudorandomized item order were assigned to the participants. Therefore, items were numbered and then randomized using a random number generator (www.random.org). After that, we manually adapted the resulting random order because of task-related issues. When a test trial followed immediately after a break, we changed the order and put a filler item instead (see Supplementary Table 2 for the item composition). In total, this experiment lasted around 10 min.

### Comprehension Data Analysis

**Figure 2:**
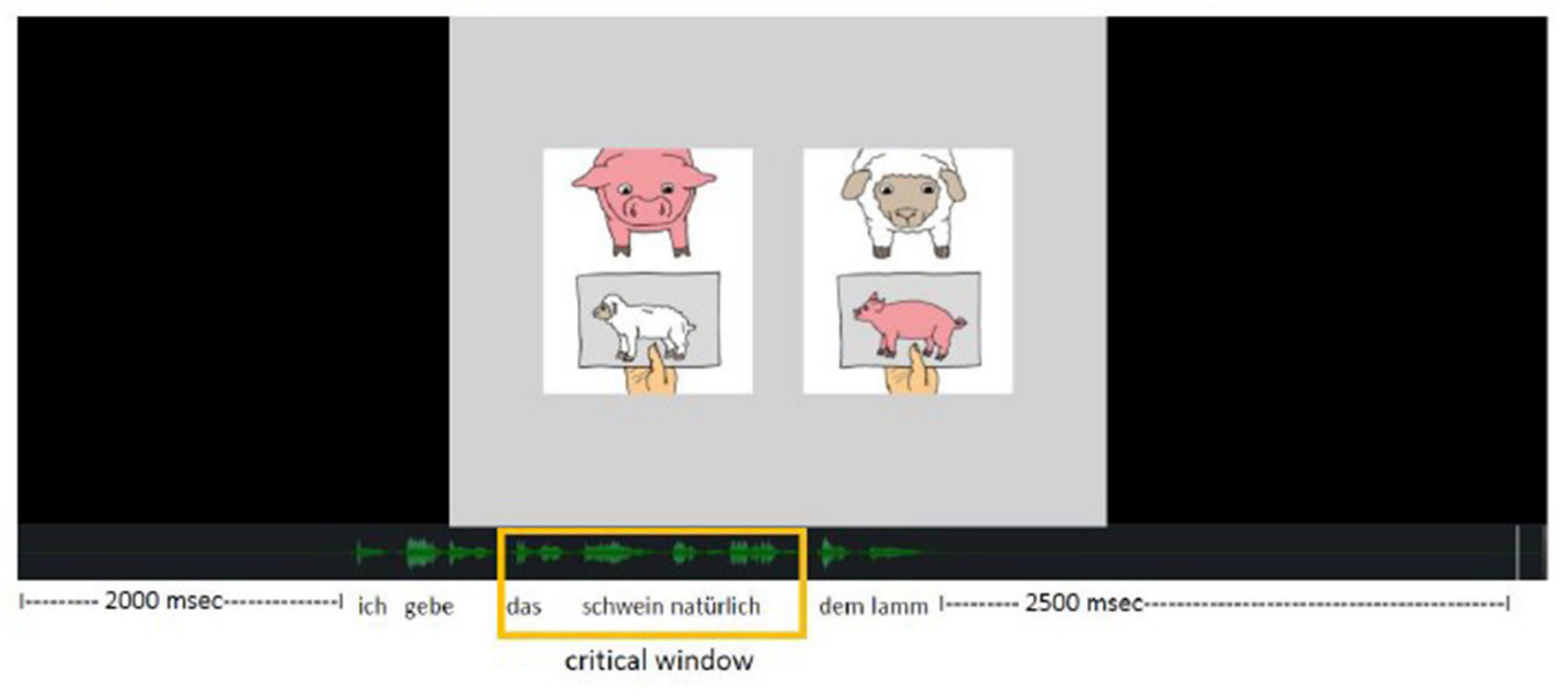
Critical Window for analysis of the reaction times in the sentence-picture matching task. Item example “ich gebe das schwein natürlich dem lamm” (“of course, i give the pig to the lamb”).

We analyzed accuracy of sentence-picture matches and RTs. For RTs, the annotation capture plugin of the software Pupil Labs was used. This plugin allows for labeling timestamps (“L” for left picture, “R” for right picture). These labels are created by pressing their respective hotkey (as for the sentence-picture matching task, we chose “a” for left and “6” for right). Regarding the RT analysis, we defined critical windows to investigate how fast participants would react after hearing the first case marking (see Figure 2). RTs were then calculated by subtracting the timestamp of the critical window's starting point from the timestamp of the label L/R. As not all of the items had precisely the same length, critical windows were defined individually for each of the 20 trials by using the software Audacity® version 2.2.2 (iWeb Media, Ltd., Birkirka, Malta). Accuracy was analyzed by comparing the participants' response (L/R) and the correct target response.

Regarding predictions, we followed Schlenter (2019, p. 2) in her assumption that “only effects visible prior to the onset of the critical perceptual input are taken as effects of prediction” in contrast to later effects that may reflect rapid integration rather than prediction. As this study instrumentalizes RTs as indicators of anticipation, we defined the time between the offset of the object's article and the onset of the second object as the critical window. Trials in which participants reacted before the onset of the second object in this critical window were classified as “predictions.” Trials in which participants reacted after the offset of the critical window were classified as “no predictions.” Since not all trials have precisely the same length, the reaction time at the participants' disposal before hearing the second object varies between trials (*min* = 1,471 ms, *max* = 1,999 ms). Therefore, prediction ratings were scored individually for each item.

Data were analyzed using linear mixed-effects models (LMMs), assuming a Gaussian distribution for interval-scaled RTs and GLMMs with a logit link function for binary accuracy with lme4 package (Bates et al., 2015) in R version 3.6.2 (R Core Team, 2020) and RStudio version 1.2.5033 (RStudio Team, 2020). For the analysis of RTs, erroneous trials were excluded. Then, we set up a model space with all reasonable models, including a null model, just including *Participant* as a random intercept. Models were compared using the AIC. For the purpose of model comparisons, Maximum Likelihood estimation method was used. The statistics of the winning model (= lowest AIC) are reported in full in the text.

### Comprehension Results

#### Comprehension Accuracy

The results of the participants' accuracy scores in the sentence-picture matching task are listed in Table 5. As can be seen, with means >95% in every group and both word orders, accuracy scores are consistently high.

**Table 5:** Monolinguals' and bilinguals' accuracy in the sentence–picture matching task.

	**IO-DO**	**DO-IO**	**Total**
**Monolinguals**			
Mean (%)	97.3	97.3	97.3
SD (%)	5.9	5.2	3.7
Range (%)	80–100	80–100	90–100
**Bilinguals**			
Mean (%)	96.7	95.6	96.1
SD (%)	8.2	10.1	7.6
Range (%)	70–100	60–100	75–100
Mann-Whitney-U-test	*p* > 0.05	*p* > 0.05	*p* > 0.05

To evaluate whether we should include a factor *Group* dissociating

- monolingual vs. bilingual participants only,
- participants according to their age of German onset: from birth as L1 (monolinguals), from birth as 2L1, early L2 onset, late L2 onset,
- participants based on the L1 case systems similar or different to German: same (monolinguals), similar, different,

we again first compared three GLMMs including different versions of *Group* as a fixed-effects factor, plus random-effects factor *Participant* as random intercept. The results were clear: The differences were negligible (AICs: 1534.87 vs. 1534.88 vs. 1534.90). We decided to continue with version (a) of factor *Group*, because of its lowest AIC.

Then, to evaluate the different influences of *Group* {monolingual, bilingual} and *WordOrder* {IO-DO, DO-IO}, we set up different models as described in Table 6 and compared them using the AIC. As to be expected from the ceiling performance of both mono- and bilinguals (i.e., above 90%; see Table 5), no model performed better than the null model M0 (see Table 6).

**Table 6:** Model space for accuracy and RTs in the comprehension task.

**Model**	**Formula**	**AIC**	
		**y = accuracy**	**y = RT**
M0	y ~ 1 + (1|Participant)	**232.33**	1435.43
M1	y ~ 1 + Group + (1|Participant)	234.22	1437.37
M2	y ~ 1 + WordOrder + (1|Participant)	233.32	**1429.75**
M3	y ~ 1 + Group + WordOrder + (1|Participant)	235.20	1431.68
M4	y ~ 1 + Group + WordOrder + Group*WordOrder + (1|Participant)	237.16	1432.98

*Formulas for all models with AICs, i.e., estimators of model fit for the sentence-picture matching task. The winning models' AICs are printed in bold font*.

Thus, regarding the accuracy in the sentence-picture matching task, we did not find any differences aside from the ones that were due to inter-individual differences based on random-effects factor *Participant* [odds ratio of intercept = 68.52, 95% CI = [28.03, 167.53], z = 9.27, *p* < 0.001].

#### Reaction Times

**Figure 3:**
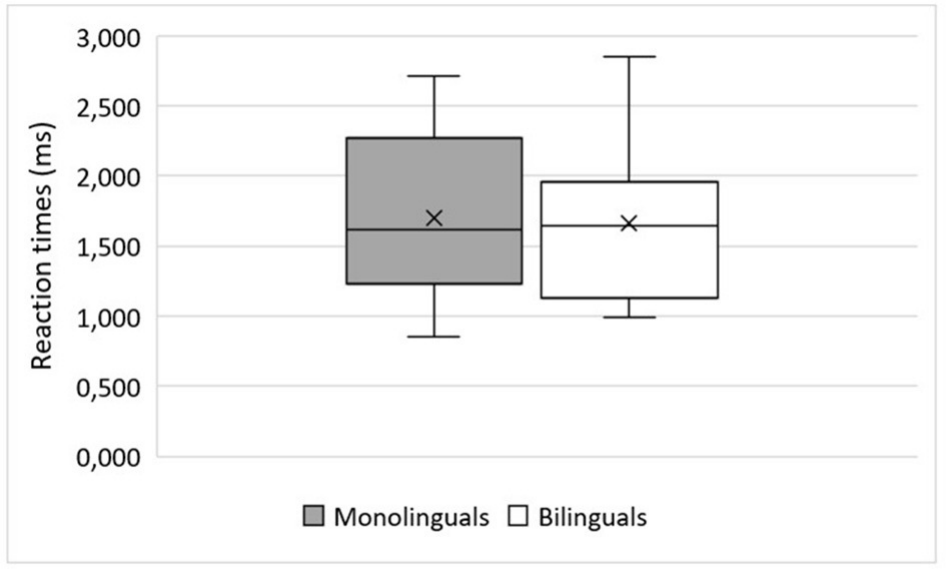
Reaction times after hearing the onset of the first object in the sentence-picture matching task in monolingual and bilingual speakers. x = mean (mean monolinguals = 1,700 ms, SD = 0,561 ms; mean bilinguals = 1,663 ms, SD = 0,562 ms).

Reaction times were similar in monolinguals and bilinguals (see Figure 3). For the analysis, we again compared three LMMs including different versions of *Group* as a fixed-effects factor, plus random-effects factor *Participant* as random intercept. The comparison of the monolingual/bilingual vs. AOO vs. case marking system of L1 models revealed that AOO was the slightly better estimate (AICs: 1437.41 vs. 1437.37 vs. 1437.40). Again, the differences in AICs are marginal. Nevertheless, we continued by modeling *Group* as AOO.

Then, to evaluate the different influences of *Group* {monolingual, bilingual} and *WordOrder* {IO-DO, DO-IO}, we set up different models as described in Table 6 and compared them using the AIC. As can be seen from Table 6, model M2 was the winning model. Random intercept for *Participant* was significant [β = 1.728, SE = 0.086, 95% CI = [1.56, 1.90], *t*(47.99) = 20.117, *p* < 0.001] and fixed-effects factor *WordOrder* was highly significant [β = −0.098, SE = 0.035, 95% CI = [−0.17, −0.03], *t*(808.19) = −2.78, *p* = 0.006].

Mean RT for IO-DO (1.637 s, SD = 0.726 s) was smaller than for DO-IO (1.723 s, SD = 0.773 s), displaying a typical word-order effect.

#### Anticipation of the Second Object

Regarding anticipatory reactions, we evaluated the influence of *Group* and *WordOrder* on whether participants were able to anticipate the upcoming second object. Only correct responses were analyzed. First, we assessed how to best model *Group* by comparing the three different assessments as before. Again, all model fit estimates (AICs) were relatively similar (monolingual/bilingual = 830.12, AOO = 830.21, case similarity = 830.23). As the simple differentiation only based on mono- vs. bilingual was the best fit, we used this for Group.

We then assessed which GLMM fit the data best with regard to whether *WordOrder* and *Group* had explanatory value for anticipation. The full model space is provided in Table 7. The model that fit the data best was the null model M0. However, even random intercept for *Participant* was not significant [odds ratio = 1.44, 95% CI = [0.72, 2.90], *p* = 0.302].

**Table 7:** Model space for anticipations of the second object in the comprehension task.

**Model**	**Formula**	**AIC**
M0	Anticipation ~ 1 + (1|Participant)	**828.24**
M1	Anticipation ~ 1 + Group + (1|Participant)	830.12
M2	Anticipation ~ 1 + WordOrder + (1|Participant)	829.71
M3	Anticipation ~ 1 + Group + WordOrder + (1|Participant)	831.59
M4	Anticipation ~ 1 + Group + WordOrder + Group*WordOrder + (1|Participant)	833.02

*The winning model's AIC is printed in bold font*.

Thus, neither *Group* nor *WordOrder* appear to influence the number of anticipated second objects.

**Figure 4:**
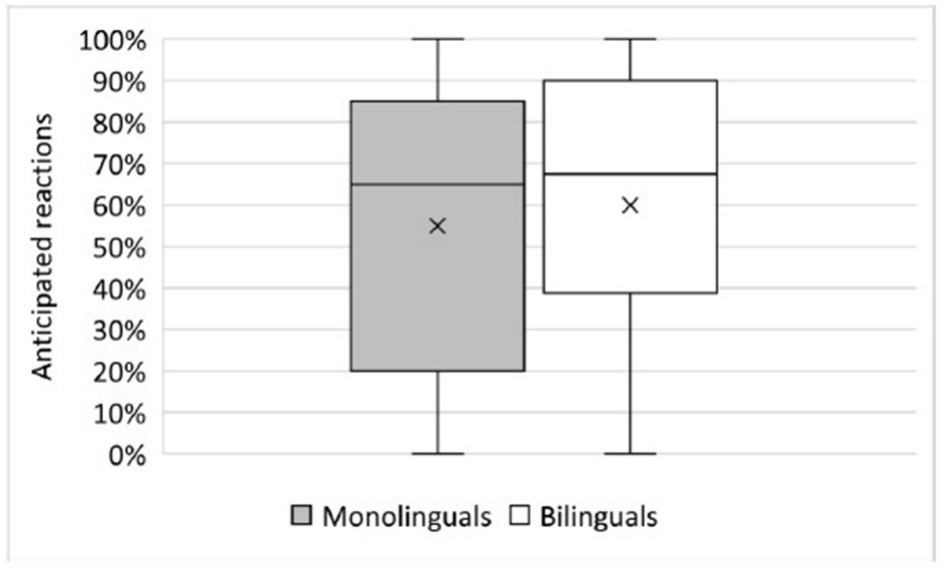
Proportion of anticipatory reactions (before hearing the second object) in the sentence-picture matching task in monolinguals and bilinguals. x = mean.

In accordance with this, Figure 4 shows that no significant differences were found between the mean percentage of anticipations after hearing the first object in monolinguals (mean = 55.0%, SD = 34.1%) and bilinguals (mean = 60.0%, SD = 34.2%; Mann-Whitney U: U = 559.5, p =0.541). Thus, bilinguals predicted the second object to the same extent, that is, in more than 60% of all items, as monolinguals.

Lastly, there were no significant correlations between any of the tested WM categories and overall rate of anticipatory reactions (see Table 8).

**Table 8:** Results of correlation analyses between working memory (forward [FW] and backward [BW] digit span) and predictive processing (in percentage of anticipatory reactions).

	**Monolinguals**	**Bilinguals**
Digit span FW–Anticipatory reactions (%)	*r* = 0.02	*r* = 0.44
	*p* > 0.05	*p* > 0.05
Digit span BW–Anticipatory reactions (%)	*r* = −0.14	*r* = 0.06
	*p* > 0.05	*p* > 0.05

## Study 3 – Pupillometry

### Pupillometry Study Design

Pupil data were tracked by a Pupil Labs eye tracker at 200 Hz (*Pupil Core*, Pupil Labs). We used a 9-point eye tracker calibration before the pupillometry task started. To avoid confounding implicit pupil measurements with a participant's explicit action, i.e., manual response, the participant's task was simply to listen to the auditory stimulus. To minimize reflexive reactions of the pupil diameter to changes in luminance, we kept the displayed colors constant. Therefore, the monitor displayed a black fixation cross on a gray background while auditory stimuli were played. After a block of five trials, there was either a comprehension question to check for the participants' attention (like e.g., “Is today Wednesday?”) or pause with a picture of a forest to relax the eyes. The only explicit reaction that was required by the participants throughout the experiment was to press a button (yes/no) to answer these comprehension questions. Throughout the presentation of the forest picture, the participants were told to do whatever they like to relax the eyes (blink, look at the picture, or look away). The stimuli following the comprehension questions and the stimuli following the relax pictures were fillers that were not analyzed.

The experiment contained 30 test items and 20 filler items such as *das schaf frisst das gras* (“the sheep eats the grass,” see Supplementary Table 3 for further examples). The test items were constructed similarly to the items from the comprehension task and contained 10 items per condition (A, B, C, see Example 11) resulting in a total amount of 30 grammatical stimuli (condition A + fillers) and 20 ungrammatical stimuli (conditions B and C). Conditions contained gradually violated grammars: Whereas condition A contained grammatical items (see Example 11a), condition B contained an accusative overgeneralization in the dative context (see Example 11b). This equals the prefinal acquisition step toward target ditransitive production in child acquisition. Condition C contained no determiners at all (see Example 11c), which equals the first step in the acquisition process. Condition B therefore was labeled “slightly ungrammatical” and condition C was labeled “strongly ungrammatical.” In total, the pupillometry experiment lasted about 9 min. 
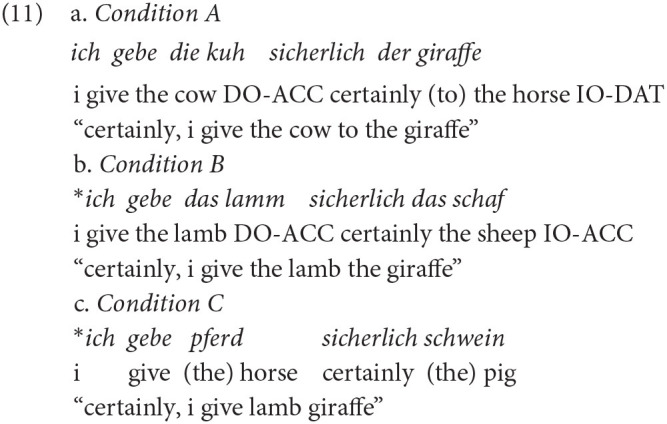


### Pupillometry Data Analysis

Pre-processing was accomplished using the package gazeR (Geller et al., 2020) in R (version 1.4.1106, R Core Team, 2020). Samples 100 ms prior and 100 ms after a blink were coded as missing and were linearly interpolated. After the interpolation process, artifacts were removed based on the median absolute deviation (see Geller et al., 2020 for details). Such artifacts stem from quick changes in pupil size. In order to smooth the pupil time course, we passed a 5-point moving average over the data. In order to account for spontaneous variation in pupil size, we baseline-corrected the pupil diameter for each trial individually. Therefore, we determined a baseline of 1,000 ms prior to audio onset. Mean pupil diameter from this baseline was then subtracted from all pupil data points of the respective trial. A time window of 3,500 ms starting from the first violation (i.e., the onset of the first object) was selected for analysis. Moreover, data were filtered for intra-individual outliers by filtering all data points with a pupil diameter +/– 2.5 SD above/below the mean.

In the absence of a “field-standard statistical approach” (Geller et al., 2020, p. 2251) to analyze pupil data, we decided to analyze the pupil dilation trajectories directly instead of extracting peak amplitudes and latencies, as recommended by van Rij et al. (2019). We therefore applied a non-linear regression analysis, i.e., generalized additive mixed modeling (GAMM). To this end, we used mgcv package version 1.8.36 (Wood, 2017; van Rij et al., 2019) in R version 4.0.4 (R Core Team, 2020) and RStudio version 1.4.1106 (RStudio Team, 2020).

### Pupillometry Results

To find the best model fitting the data, we set up a model space including different combinations of fixed-effects factors and random smooths as elaborated below. The full model space is provided in Table 9, where *y* is the baseline-corrected, outlier-corrected (+/– 2.5 SD of the mean) pupil dilation. All models were estimated using Maximum Likelihood estimation.

**Table 9:** Model space for pupil dilation in the pupillometry task.

**Model**	**Simplified R formula**	**AIC**
M00	y ~ 1 + s(x, y) + s(time, participant)	272547.51
M0	y ~ 1 + s(x, y) + s(time, subject) + s(time, trial)	268361.61
M1	y ~ 1 + violation + s(x, y) + s(time, by = violation) + s(time, subject) + s(time, trial)	148714.02
M2	y ~ 1 + group + s(x, y) + s(time, by = group) + s(time, subject) + s(time, trial)	149523.24
M3	y ~ 1 + viogr + s(x, y) + s(time, by = viogr) + s(time, subject) + s(time, trial)	**147441.16**

*The winning model's AIC is printed in bold font*.

Furthermore, all models include a covariate smooth term for gaze direction s (x,y), because the measured pupil dilation is confounded with it. Depending on the angle, the pupil appears oval instead of round, leading to an underestimation of the pupil size. The null model M00 further includes a random smooth for *Participant* over *Time s* {time, participant} to model the variance of the pupil response over time that is only due to inter-individual differences. We chose to also estimate a second null model M0, which also included a random smooth *s* {time, trial}to account for trial-wise variation over time. Because M0 was the better null model, we included both random smooths in all models of interest (M1–M3, Table 9).

Models M1–M3 further included either a fixed-effects factor to model main effect of *Violation* {A, B, C}, *Group* {monolingual, bilingual}, or both *VioGr* {A-monolingual, A-bilingual, B-monolingual, B-bilingual, C-monolingual, C-bilingual}. Due to the nature of GAMMs, an interaction term cannot be included as one would do for LMMs. Thus, we included a factor *VioGr* combining all levels of *Violation* and *Group*. The best fitting model by far was M3, the one including the factor *VioGr*.

**Figure 5:**
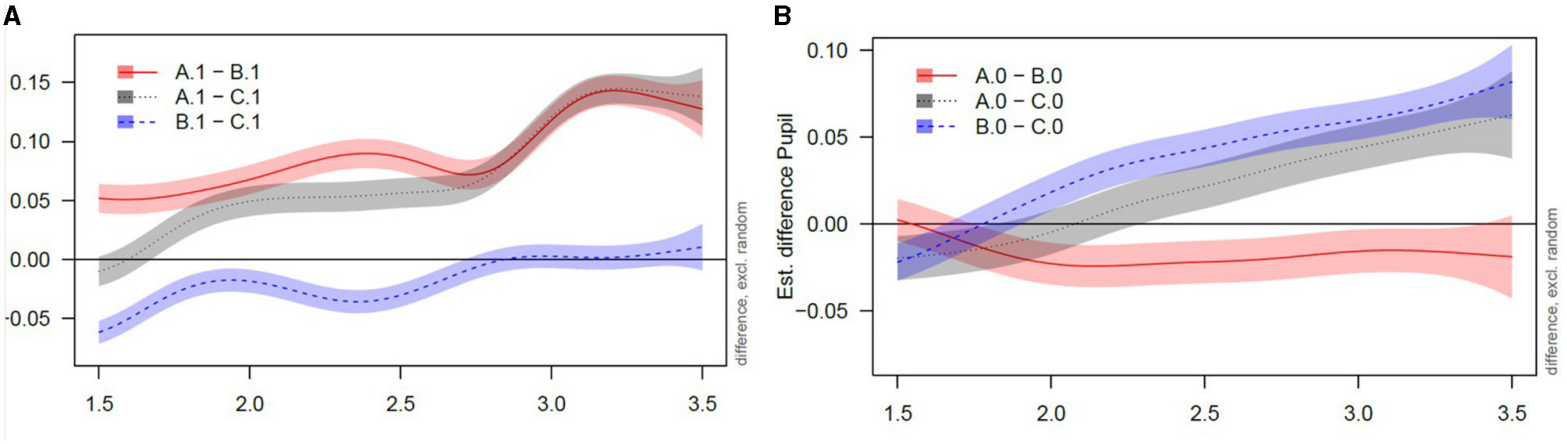
Monolinguals' and bilinguals' pupil dilation in the pupillometry task. Pupil dilation within the analysis window of 3,500 ms (time zero starting at the onset of the first object) with plotted differences between conditions. Curves around 0.00 indicate no difference between the plotted conditions (e.g., between A0 and B0). Curves deviating from 0.00 indicate differences between the plotted conditions in the pupil dilation trajectory. A1 = condition A (grammatical) in monolingual data; A0 = condition A (grammatical) in bilingual data; B1 = condition B (slightly ungrammatical) in monolingual data; B0 = condition B (slightly ungrammatical) in bilingual data; C1 = condition C (strongly ungrammatical) in monolingual data; C0 = condition C (strongly ungrammatical) in bilingual data. Panel **(A)**: Monolinguals. Panel **(B)**: Bilinguals.

In this task the pupil dilation is expected to change with violation (condition B and C vs. condition A). As to be expected from the winning model M3, excluding random effects of *Participant* and *Trial*, pupil dilation varied as a function of *Violation* {A, B, C} and *Group* {monolingual, bilingual}. This is depicted in Figure 5.

As Figure 5 illustrates, both groups react differently to different conditions. As suggested in van Rij et al. (2019), we inspected the model's estimates of the differences between the conditions visually and therefore decided to interpret the time window where curves of differences obviously change (from around 1.50 s after the time zero for both groups, see Figure 5). To be temporally exact, for monolinguals, the difference of the pupil dilation for grammatical sentences (A) and slight violations of type B was significant between 1.50 and 3.50 s. Similarly, pupil dilation in response to grammatical sentences (A) and violations of type C showed significant differences between 1.77 and 3.50 s. Both violation conditions (B vs. C) on the other hand differed significantly between 1.50 and 2.83 s and then converged. Contrarywise, bilinguals showed significant differences between grammatical sentences (A) and violations of type B between 1.67 and 3.50 s, and for A and C, a difference was observed only later between 2.68 s and 3.50 s. In bilinguals, the two violation conditions B and C differed between 2.02 and 3.50 s. Thus, B and C did not converge as in monolinguals. Therefore, the main difference between monolinguals and bilinguals is the bilinguals' relatively late reaction on strongly ungrammatical sentences (2.68 s in bilinguals vs. 1.77 s in monolinguals). The differences in pupil dilation time courses (as a covert or implicit response) for mono- and bilinguals are especially interesting in light of the missing behavioral differences, that is, of the overt response. This will be discussed below.

## Discussion

This paper aimed to investigate monolingual and bilingual adult speakers of German with respect to their linguistic performance regarding production, comprehension, and implicit sensitivity to grammatical violations of the morphosyntactic complex double-object construction with ditransitive verbs. Therefore, we developed three experiments in which we elicited ditransitive structures (study 1), assessed accuracy and RTs in a sentence-picture matching task (study 2), and investigated sensitivity to grammatical violations via pupillometry (study 3).

In comprehension and production, bilinguals exhibited abilities that were comparable to monolinguals' in all aspects. Regardless of the AOO (0;0 for 2L1, 3;0–4;0 for eL2 and 6;0–13;0 for late L2), all bilinguals performed at ceiling in production and comprehension tasks. The reported overgeneralisations of accusative in dative contexts in production were negligible and can be interpreted as performance errors. However, since *WordOrder* had a descriptive, but non-significant influence on the production of the dative it would be interesting to see, whether the effect might reach significance, when performance is not at ceiling.

The ability to anticipate upcoming input, i.e., using the case marking of the first object (dative marking in default word order and accusative marking in marked word order) to anticipate the upcoming input in bilinguals was comparable to that in monolingual speakers. This is in accordance with Schlenter's (2019) findings and contradicts the RAGE hypothesis, which states that bilinguals have a reduced ability to predict upcoming input (Grüter et al., 2014). The variation in RTs within the bilingual group could not be explained by different AOOs. Thus, we did not observe that 2L1 speakers reacted faster than monolinguals (as it was the case in Desideri and Bonifacci, 2018). However, a bigger sample size is needed to generalize these preliminary results. A more trivial explanation for the lacking difference in our findings between monolinguals and bilinguals may be that the task was too easy for the adult participants to reveal subtle processing differences, owing to ceiling effects. This potential limitation could be addressed in follow-up studies that employ tasks that are more difficult. However, the aim of such a study would stand to question. There are probably no real-world implications if a task has to be extremely difficult for differences to emerge between bilinguals and monolinguals.

As can be seen in the huge variability in monolingual and bilingual adults regarding anticipated reactions, the methodological set-up of this study limits definite conclusions about the underlying predictive ability. Nevertheless, the data show comparable levels of anticipated reactions for mono- and bilinguals, supporting the overall impression of bilinguals' native speaker competence. This is in line with Halliday's (1975) definition of a native speaker as someone who is able to predict what the other person is going to say and therefore being able to anticipate upcoming input. Furthermore, we found a word order effect in the comprehension task that concerned the strategy of “IO-first.” There was a word order bias in favor of the IO-DO order in the sense that speakers reacted significantly faster in trials with unmarked IO-DO word order than in the marked DO-IO word order. This finding is in line with Kholodova and Allen (in press) report on the productive preference of IO-DO word order in adult native German speakers. In accordance with the N1 bias found for subjects, where children up to puberty implicitly prefer interpreting the first NP in an utterance as the subject (Lidzba et al., 2013), this can be interpreted as a processing strategy. The investigated speakers implicitly assume that the first NP is the subject and the second NP is the recipient (i.e., the IO in ditransitives), which in most naturally occurring cases leads to the correct utterance interpretation. Applying this strategy may enhance utterance interpretation. Both, monolingual and bilingual speakers show this robust word order effect in the sentence-picture matching task.

The analysis of the participants' pupil data revealed a clear difference between the implicit response to grammatical (condition A) compared to slightly ungrammatical (condition B) and strongly ungrammatical sentences (condition C) in both, monolinguals and bilinguals. Taking, for instance, Chomsky's relation between native speaker and a grammatical sentence as a basis, the participants investigated here can be claimed highly competent since they were able to identify ungrammatical utterances implicitly and thus provide valid implicit grammaticality judgements on their language on the basis of intuitive knowledge of the grammatical sentence (Chomsky, 2002).

However, statistical analyses revealed that pupil dilation varied not only as a function of *Violation* {A, B, C} but also as a function of *Group* {monolingual, bilingual}. The main difference between monolinguals and bilinguals in the timing of pupil reactions concerns the difference between A and C, i.e., the grammatical condition and the strongly ungrammatical condition. Here, monolinguals react faster to the grammatical violation than bilinguals, demonstrated by the differences between A and C that become significant earlier in monolinguals than in bilinguals. The bilinguals' delayed response when compared to monolinguals could be due to higher processing efforts. This interpretation would be in line with Fernandez (2016) who interprets longer peak latencies (i.e., the time until the pupil is maximally dilated) as measures for higher processing effort.

Overall, despite bilinguals' comparable production and comprehension ability evident from their behavioral responses, the pupil dilation data revealed a temporal difference in violation detection for strong grammatical errors. This in turn indicates a subtle difference between monolingual and bilingual speakers' reactions to these strong grammatical violations. This could be interpreted as confirming Paradis' (2016, 2019) findings of bilingual speakers being typically indistinguishable from monolingual speakers in conversation, while they differ when it comes to grammaticality judgement tasks regarding complex morphosyntax in adulthood. However, our data revealed bilinguals to be able to identify these violations but with a different pace than monolinguals. Therefore, we argue for an intact although somewhat delayed implicit identification ability of grammatical violations and assume that there is no real-life effect of this delay in the millisecond range.

However, a further methodological note is important at this point. Even though the AIC differences were rather small, the AICs for three of the models we calculated for production and comprehension data indicated that some of the variance is better explained by a Group variable that does not only differentiate between monolinguals and bilinguals, but that differentiates bilinguals further based on their AOO. Thus, with a larger sample size and more data from highly proficient bilinguals with different AOOs, we might be able to detect a potential AOO effect that is concealed in our data by the small^4^ sample sizes of bilingual subgroups with different AOOs. This is up to future research.

### Concluding Remarks

Overall, the results of this pilot study demonstrate high proficiency in production as well as comprehension in the studied group of bilingual adult speakers of German regardless of their (2)L1 and AOO, evident in their comparable ceiling performance in production and comprehension, as well as their high sensitivity to grammatical violations in German utterances. We therefore conclude that at this proficiency level, AOO and cross-linguistic influence may not affect production and comprehension abilities in bilingual adult speakers of German. The documented high proficiency in all domains is in line with Hartshorne et al. (2018), who proposed the sensitive period up to age 17. We did not find support for a turning point of grammar learning abilities at a younger age (contrasting Meisel, 2018). Regarding ultimate attainment, this indicates that even after a relatively late AOO (13 years), complex morphosyntactic structures such as ditransitives can be mastered in production and comprehension to a monolingual and 2L1 native-speaker degree. Conversely, bilinguals have the advantage of having acquired an additional language while showing the same explicit and implicit grammatical knowledge of complex structures like monolinguals. However, a possible limitation is the small sample size of the current study. The results do not imply that every eL2 child can master case marking in ditransitives, but they at least provide evidence that early and even late L2 learners of German can master these rather complex structures in morphosyntax, and thus reach a competence level comparable to monolinguals or 2L1 speakers.

In line with psycholinguistic research on early bilingualism, we included a control group of monolingual speakers in the present study. Our aim was to challenge the idealized monolingual L1 competence as the native speaker norm and evaluate the validity of this assumption. Our findings provide evidence for a new perspective on the term “native speaker.” Here, our data revealed that even so-called near-native speakers with AOOs later than 6 years could show high proficiency throughout. Characteristics previously attributed to native speakers (i.e., prediction of upcoming input in Halliday, 1975, and valid grammaticality judgements by Chomsky, 2002) were also found to be true for the bilingual speakers investigated in the present study. Therefore, the connotation of the term “native” with high proficiency and “non-/near-native” with lower proficiency appears to be misleading. The term “near-native” suggests that someone who is not born in the country of the L2 is only near to the competence of a native speaker (see Bylund et al., 2021), whereas “native” is equated with monolingualism, and monolingualism in turn is equated with the highest proficiency. However, the huge variation in the RTs and in the pupillometry measures of the investigated monolinguals of our study convey a different picture: Even within highly proficient monolingual native speakers, there is considerable variance with regard to language skills. If the explicit and implicit competence of bilingual speakers (who at random have not been raised with the language under investigation from birth) fall within this spectrum of high proficiency, then it is not reasonable to call them near-native, but instead to focus on their competences, which may very well be at monolingual native level. The term “near-native” is connoted with imperfection that could not be documented in our investigated bilingual speakers.

The term “native speaker” is misleading in the sense that being raised monolingually does not mean being perfect in every subtle part of one's own language (see high in-group variance in RTs). On the other hand, acquiring an additional language or being raised bilingually does not mean that someone is not able to achieve high competence, also in subtle and implicit measures. We therefore call for a different perspective on someone's language competence other than to tie it invariably to the place of birth and upbringing and the amount of languages that have been acquired.

To this end, the two “groups” (monolinguals and bilinguals) that we compared and contrasted in our study can be collapsed in to one group: In fact, we see one group of highly proficient speakers of German with high performances on different explicit and implicit tasks that concern complex morphosyntax. With the add-on of some speakers who are additionally able to speak another language, representing a personal advantage for these speakers.

## Data Availability Statement

The raw data supporting the conclusions of this article will be made available by the authors, without undue reservation.

## Ethics Statement

Ethical review and approval was not required for the study on human participants in accordance with the local legislation and institutional requirements. The patients/participants provided their written informed consent to participate in this study.

## Author Contributions

A-LS developed the research ideas, the design of the study, and wrote the first draft of the manuscript. A-LS collected the data, with the help of JK. A-LS, JK, and TL performed the statistical analysis and created the visualizations. GU and JK wrote sections of the manuscript. All authors contributed to manuscript revision and approved the submitted version.

## Conflict of Interest

The authors declare that the research was conducted in the absence of any commercial or financial relationships that could be construed as a potential conflict of interest.

## Publisher's Note

All claims expressed in this article are solely those of the authors and do not necessarily represent those of their affiliated organizations, or those of the publisher, the editors and the reviewers. Any product that may be evaluated in this article, or claim that may be made by its manufacturer, is not guaranteed or endorsed by the publisher.

## Footnotes

^1^Scherger, A.-L., Kizilirmak, J. M., and Folta-Schoofs, K. (submitted). Ditransitive structures in child language acquisition: an investigation of production and comprehension in children aged five to seven. *J. Child Lang*.

^3^The AIC is an estimation of the relative quality of statistical models, considering a trade-off between the goodness of fit and simplicity of the model. Generally speaking: The model with the smallest value within a comparison fits the data best.

^4^It should be noted that the study was not designed to further split bilinguals based on AOO. This modeling was an exploratory addition.
