# Corrigendum: The Bilingual Native Speaker Competence: Evidence From Explicit and Implicit Language Knowledge Using Elicited Production, Sentence-Picture Matching, and Pupillometry

**DOI:** 10.3389/fpsyg.2021.790449

**Published:** 2021-11-10

**Authors:** Anna-Lena Scherger, Gianna Urbanczik, Timon Ludwigs, Jasmin M. Kizilirmak

**Affiliations:** ^1^Department of Rehabilitation Sciences, Language & Communication, TU Dortmund University, Dortmund, Germany; ^2^University of Hildesheim, Institute for Psychology, Neurodidactics & NeuroLab, Hildesheim, Germany; ^3^German Center for Neurodegenerative Diseases, Cognitive Geriatric Psychiatry, Göttingen, Germany

**Keywords:** language production, language comprehension, ditransitives, implicit and explicit knowledge, article omission, pupillometry

In the published article, there was an error in affiliation 2. Instead of “University of Hildesheim, Institute for Psychology, Hildesheim, Germany”, it should be “University of Hildesheim, Institute for Psychology, Neurodidactics & NeuroLab, Hildesheim, Germany.”

Additionally, in the original article, there was an error in the Acknowledgments section. In the original version, it read “We thank Tom Fritzsche for helpful comments on the pupillometry experiment setup and Prof. Kristian Folta-Schoofs for allowing the usage of his lab.”

A correction has been made to *Acknowledgments*. The corrected paragraph is shown below.

Acknowledgments

We acknowledge financial support by Deutsche Forschungsgemeinschaft and TU Dortmund University within the funding programme Open Access Publishing. We acknowledge financial support by the University of Hildesheim for a student assistant. We thank Tom Fritzsche for helpful comments on the pupillometry experiment setup and Prof. Dr. Kristian Folta-Schoofs for allowing the usage of his lab and equipment. For their help with data collection, we thank Klara Tittelbach-Helmrich and David Mietzner. We further thank Hannes Elfers and the Elisabethschule in Hildesheim for their invaluable help with data collection and Julia Diedrich for illustrating the visual stimuli. Above all, we thank our participants.

Unfortunately, in the original article, a number of references also contained errors as listed below.

For Kauschke and Siegmüller (2010), the city of the publisher was incorrect. It was incorrectly written as Kauschke, C., and Siegmüller, J. (2010). *Patholinguistische Diagnostik bei Sprachentwicklungsstörungen (PDSS) [Patholinguistic Assessment of Developmental Language Disorders in German], 2nd Edn*. Amsterdam: Elsevier. The corrected reference is shown below.

The reference for Preuschoff et al. (2011) contained an incorrect name for one author. It was incorrectly written as Preuschoff, K., Hart, B. M., and 't Einhäuser, W. (2011). Pupil dilation signals surprise: evidence for noradrenaline's role in decision making. *Front. Neurosci*. 5:115. 10.3389/fnins.2011.00115. The corrected reference is shown below.

The reference for Schmitz (2006) incorrectly contained a URL. It was incorrectly written as: Schmitz, K. (2006). “Indirect objects and dative case in monolingual German and bilingual german/romance language acquisition,” *in Studies in Language Companion Series: v. 75. Datives and Other Cases: Between Argument Structure and Event Structure*, eds D. P. Hole, W. Abraham, and A. Meinunger (Ahttp://en.wikipedia.org/wiki/Amsterdam msterdam: J. Benjamins), 239–268. The corrected reference is shown below.

The authors apologize for these errors and state that they do not change the scientific conclusions of the article in any way. The original article has been updated.

## Publisher's Note

All claims expressed in this article are solely those of the authors and do not necessarily represent those of their affiliated organizations, or those of the publisher, the editors and the reviewers. Any product that may be evaluated in this article, or claim that may be made by its manufacturer, is not guaranteed or endorsed by the publisher.
