# Supplementary material for: The Bilingual Native Speaker Competence: Evidence From Explicit and Implicit Language Knowledge Using Elicited Production, Sentence-Picture Matching, and Pupillometry
Source: Front Psychol. 2021 Sep 16;12:717379. doi: 10.3389/fpsyg.2021.717379 (PMC8483243; doi:10.3389/fpsyg.2021.717379)
Supplement: Supplementary file 1 [file Data_Sheet_1.pdf]

Supplementary Material

Item lists

The following item lists serve the purpose of giving the reader an impression of the material by providing the first 20 items of the comprehension and the pupillometry task as well as examples of the participants' utterances in the production task. For replication purposes, the whole dataset is stored in an open repository. The link can be asked from the corresponding author.

Production task

Table A1. Examples of Utterances in the Production Task

IO-DO			DO-IO		
ich schenke	dem hund	die giraffe	ich schenke	den fisch	dem schaf
i give	(to) the dog	the giraffe	i give	the fish	(to) the sheep
'i am giving the giraffe to the dog'			'i am giving the fish to the sheep'		
ich schenke	der schnecke	die schlange	ich schenke	die ziege	dem hund
i give	(to) the snail	the snake	i give	the goat	(to) the dog
'i am giving the snake to the snail'			'i am giving the goat to the dog'		
ich schenke	dem schaf	die schildkröte	ich schenke	die maus	der schnecke
i give	(to) the sheep	the turtle	i give	the mouse	(to) the snail
'i am giving the turtle to the sheep'			'i giving the mouse to the snail'		

Comprehension task

Table A2. Insight into Item List of the Sentence-Picture Matching Task

	Target	Distractor	Condition
1	das dreieck ist schwarz<br>the triangle is black<br>'the triangle is black'	das dreieck ist blau<br>the triangle is blue<br>'the triangle is blue'	filler
2	das schaf frisst auf der wiese<br>the sheep eats on the meadow<br>'the sheep is eating in the meadow'	der hund frisst<br>the dog eats<br>'the dog is eating'	filler
3	ich schenke dem hund natürlich den fuchs<br>i give (to) the dog of course the fox<br>'I give the fox to the dog, of course'	ich schenke dem fuchs den hund<br>i give (to) the fox the dog<br>'i give the to the fox the dog'	filler (masc)
4	die maus schläft<br>the mouse sleeps<br>'the mouse is asleep'	die katze schläft<br>the cat sleeps<br>'the cat is asleep'	filler
5	das viereck ist grün<br>the square is green<br>'the square is green'	der kreis ist blau<br>the circle is blue<br>'the circle is blue'	filler
6	ich gebe dem luchs sicherlich den wolf<br>i give (to) the lynx certainly the wolf<br>'I certainly give the wolf to the lynx'	ich gebe dem wolf den luchs<br>i give (to) the wolf the lynx<br>'I give the lynx to the wolf'	filler (masc)
7	ich schenke dem pferd natürlich das lamm<br>i give (to) the horse of course the lamb<br>'I give the lamb to the horse, of course'	ich schenke dem lamm das pferd<br>i give (to) the lamb the horse<br>'I give the horse to the lamb'	IO-DO
8	ich schenke das pony sicherlich dem schwein<br>i give the pony certainly (to) the pig<br>'I give the pony to the pig, of course'	ich schenke dem pony das schwein<br>i give (to) the pony the pig<br>'I give the pig to the pony'	DO-IO
9	der junge läuft neben das haus<br>the boy runs beside the house<br>'the boy is running to the side of the house'	der junge läuft neben dem haus<br>the boy runs beside the house<br>'the boy is running around next to the house'	filler
10	das mädchen hüpft im grünen wald<br>the girl hops in the green forest<br>'the girl is hopping in the green forest'	das mädchen hüpft in den grünen wald<br>the girl hops into the green forest<br>'the girl is hopping into the green forest'	filler
11	ich schenke dem pony sicherlich das schwein<br>i give (to) the pony certainly the pig	ich schenke dem schwein das pony<br>i give (to) the pig the pony	IO-DO

	‘I certainly give the pig to the pony’	‘I give the pony to the pig’	
12	das dreieck ist blau<br>the triangle is blue<br>‘the triangle is blue’	der kreis ist rot<br>the circle is red<br>‘the circle is red’	filler
13	die katze schläft im bett<br>the cat sleeps in the bed<br>‘the cat is sleeping on the bed’	die maus schläft<br>the mouse sleeps<br>‘the mouse is asleep’	filler
14	der hamster läuft<br>the hamster runs<br>‘the hamster is running’	der hamster schläft<br>the hamster sleeps<br>‘the hamster is asleep’	filler
15	das dreieck ist blau<br>the triangle is blue<br>‘the triangle is blue’	das dreieck ist schwarz<br>the triangle is black<br>‘the triangle is black’	filler
16	ich gebe das schwein natürlich dem pony<br>i give the pig of course (to) the pony<br>‘I give the pig to the pony, of course’	ich gebe dem schwein das pony<br>i give (to) the pig the pony<br>‘I give the pony to the pig’	DO-IO
17	das baby krabbelt unter das bett<br>the baby crawls under the bed<br>‘the baby is crawling under the bed’	das baby krabbelt unter dem bett<br>the baby crawls under the bed<br>‘the baby is crawling underneath the bed’	filler
18	ich gebe dem hund natürlich den fuchs<br>i give (to) the dog of course the fox<br>‘i give the fox to the dog, of course’	ich gebe dem fuchs den hund<br>i give (to) the fox the dog<br>‘I give the dog to the fox’	filler<br>(masc)
19	ich gebe das schwein sicherlich dem schaf<br>i give the pig certainly (to) the sheep<br>‘I certainly give the pig to the sheep’	ich gebe dem schwein das schaf<br>i give (to) the pig the sheep<br>‘I give the sheep to the pig	DO-IO
20	der große Kreis ist rot<br>the big circle is red<br>‘the big circle is red’	das dreieck ist blau<br>the triangle is blue<br>‘the triangle is blue’	filler

Note. This table shows the first 20 items (total number of items: *N* = 58). Target = picture that matches the audible utterance; distractor = picture that is shown on the screen next to the target picture. Masc = masculine gender.

Pupillometry task

Table A3. Insight into the Item List of the Pupillometry Task

	Item	Translation	Condition
1	das pferd läuft in den stall	the horse is running into the barn	F
2	*ich schenke pony sicherlich lamm	*i certainly give pony certainly lamb	C
3	die kuh steht auf der weide	the cow is standing on the pasture	F
4	ich gebe die kuh sicherlich der giraffe	i give the cow certainly to the giraffe	A
5	der hund läuft über die straße	the dog is crossing the street	F
	ist heute mittwoch?	is today wednesday?	question
6	der hase hoppelt über das feld	the hare is hopping across the field	F
7	das baby krabbelt unter den tisch	the baby is crawling under the table	F
8	ich gebe das pony sicherlich dem lamm	i give the pony certainly to the lamb	A
9	*ich schenke das pony natürlich das schwein	*i give the pony of course the pig	B
10	der bauer fährt mit dem trecker	the farmer drives the tractor	F
			break
11	ich gehe über die straße	i cross the street	F
12	*ich schenke das lamm sicherlich das schaf	*i certainly give the lamb the sheep	B
13	*ich gebe schwein sicherlich pferd	*i certainly give pig horse	C
14	*ich schenke lamm sicherlich pony	*i certainly give lamb pony	C
15	ich schenke dem schwein sicherlich das schaf	i give to the pig certainly the sheep	A
	ist angela merkel die derzeitige bundeskanzlerin deutschlands?	is angela merkel the current chancellor of germany?	question
16	der junge hüpft ins planschbecken	the boy jumps into the pool	F
17	*ich schenke lamm sicherlich schaf	*i certainly give lamb sheep	C
18	das baby krabbelt unter das bett	the baby crawls under the bed	F
19	*ich gebe das schwein sicherlich das pony	*i certainly give the pig the pony	B
20	*ich gebe das schaf natürlich das schwein	*i give the sheep the pig of course	B
			break

Note. F = unrelated filler; A = grammatical; B = slightly ungrammatical; C = strongly ungrammatical.
